# Supplementary material for: Chloride dynamics alter the input-output properties of neurons
Source: PLoS Comput Biol. 2020 May 26;16(5):e1007932. doi: 10.1371/journal.pcbi.1007932 (PMC7307785; doi:10.1371/journal.pcbi.1007932)
Supplement: S1 Text — (DOCX) [file pcbi.1007932.s001.docx]

## S1 Text. Converting KCC2 “pump strength” parameter from $\frac{\boldsymbol{1}}{\boldsymbol{mM} \boldsymbol{s}}$ to $\frac{\boldsymbol{mA}}{\boldsymbol{m}\boldsymbol{M}^{\boldsymbol{2}} \boldsymbol{c}\boldsymbol{m}^{\boldsymbol{2}}}$

For a cylinder, $\mathrm{volume}=L \pi r^{2}$ and $surface area=2L \pi r$, where $L$ and $r$ are the length and radius, respectively. Thus, the extrusion constant can be converted as such:

$$\begin{aligned} P_{I}=P_{mM} F\frac{\mathrm{volume}}{surface area} = P_{mM} F\frac{r}{2}\#A1 \end{aligned}$$

where $P_{I}$is the extrusion constant in $\frac{mA}{mM^{2} dm^{2}}$, $P_{mM}$ is the extrusion constant in $\frac{1}{mM s}$, $F$ is the Faraday constant in $\frac{mA s}{mmol}$.

Checking units, it is convenient to keep the units for $volume$and$surface area$,

$$\underset{P_{mM}}{\underbrace{\frac{1}{mM s}}}\underset{F}{\underbrace{\frac{mA s}{mmol}}}\underset{\frac{\mathrm{volume}}{surface area}}{\underbrace{\frac{dm^{3}}{dm^{2}}}}$$

$$\frac{1}{mM}\frac{mA}{mmol} \frac{dm^{3}}{dm^{2}}$$

Remembering that $mM=\frac{mmol}{L}=\frac{mmol}{dm^{3}}$,

$$\frac{1}{mM}\frac{mA}{\boxed{mmol}} \frac{\boxed{dm^{3}}}{dm^{2}}$$

$$\frac{mA}{mM^{2} dm^{2}}$$

For units in $cm^{2}$, as typically used in current per area, $\frac{mA}{cm^{2}}$,

$$\frac{mA}{mM^{2} cm^{2}}\frac{1}{100}$$

For $P_{mM}=0.001\frac{1}{mM s}$ and a neuron segment with $r=4 um$, $P_{I}={1.9297\times10}^{-5} \frac{mA}{mM^{2} cm^{2}}$
