# Supplementary material for: Chloride dynamics alter the input-output properties of neurons
Source: PLoS Comput Biol. 2020 May 26;16(5):e1007932. doi: 10.1371/journal.pcbi.1007932 (PMC7307785; doi:10.1371/journal.pcbi.1007932)
Supplement: S1 Fig — (PDF) [file pcbi.1007932.s002.pdf]

S1 Fig. Chloride dynamics shift the neuronal input-output function via changes to EGABA under multiple conditions.

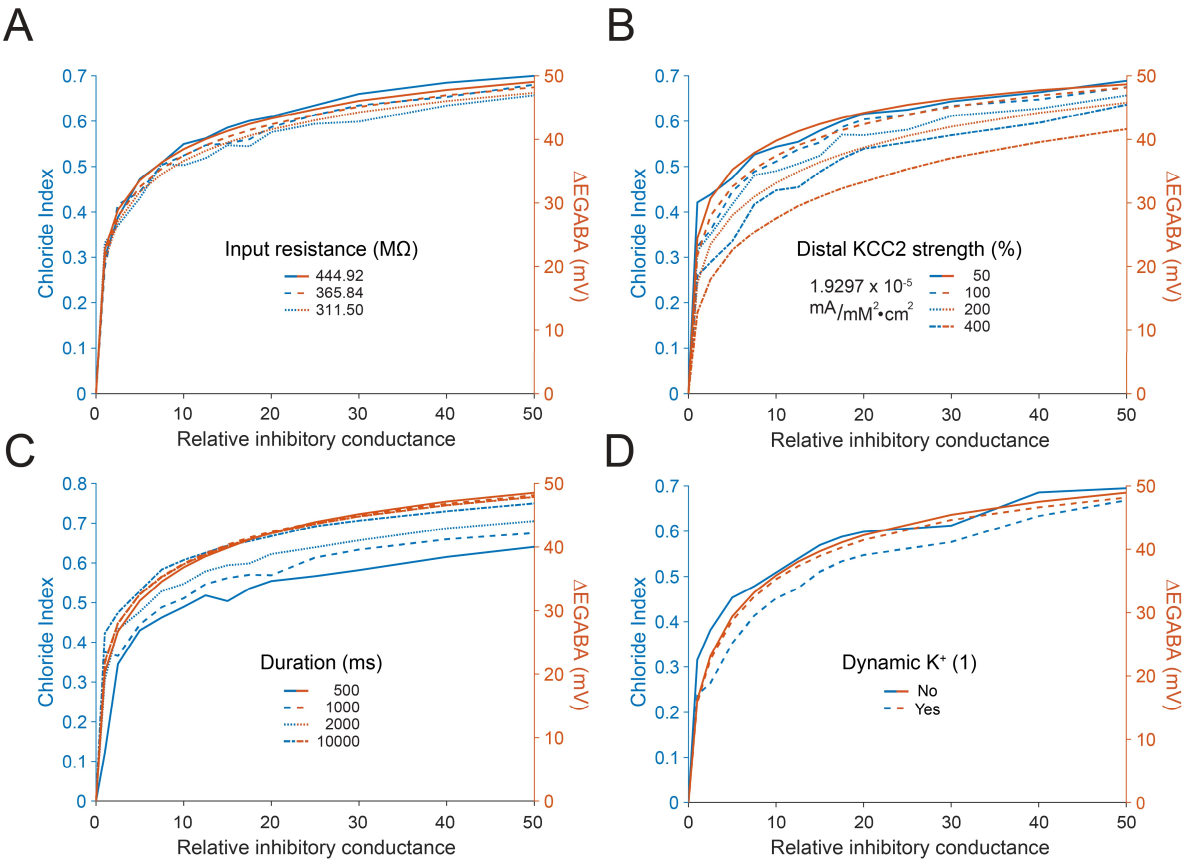

Supplementary Fig 1. Chloride dynamics shift the neuronal input-output function via changes to EGABA under multiple conditions.

Chloride index (blue, traces) and the change in dendritic EGABA (ΔEGABA, orange traces) after a 1 s simulation as a function of differing levels of peripherally targeted inhibition (as in Fig 6B and Fig 6C) under multiple conditions where different parameters of the model or simulation were adjusted: (A) Simulations where the neuronal input resistance was altered by proportionally adjusting leak conductances. (B) Simulations where distal KCC2 stength was systematically adjusted while keeping the proximal, somatic, and axonal KCC2 strength constant. (C) Simulations of differing durations demonstrate the progression of the effects of chloride dynamics on the input-output function over time. (D) Simulations with dynamic Cl^- as well as dynamic K^+ ('Yes') or static K^+ ('No'). Data from all traces were used for the summary plot in Fig 6D, Chloride index vs ΔEGABA.
