# Supplementary material for: Chloride dynamics alter the input-output properties of neurons
Source: PLoS Comput Biol. 2020 May 26;16(5):e1007932. doi: 10.1371/journal.pcbi.1007932 (PMC7307785; doi:10.1371/journal.pcbi.1007932)
Supplement: Attachment — Submitted filename: Response_to_reviewers (Currin et al).docx [file pcbi.1007932.s003.docx]

Response to reviewers

We thank the editorial team and the reviewers for their insightful comments.

*Reviewer #1: The paper combines experimental work and computer simulations to investigate the impact of fluctuations in Cl- concentration on neural signalling which is an important topic. I congratulate the authors for an interesting an well written paper. I like the concept of Cl- index.*

We thank the reviewer for their encouraging comments.

*I however had the following questions/comment*

*1) The simulations last for 1 second. If I understand correctly, intracellular Cl concentration starts at a low level and then accumulates throughout the simulations. For the results to be relevant, Cl concentration must have reached some kind of steady-state or mean steady state level by the end of the simulations. How do they authors ensure that Cl concentration has converged during the 1s timespan of the simulation? Since dynamics of ionic concentrations are typically very slow, it is not clear to me a priori that 1 second is enough? I suggest the authors increase the duration of the simulations or at least provide a convincing argument for the convergence of Cl concentration.*

The reviewer is correct in that “*intracellular Cl concentration starts at a low level and then accumulates throughout the simulations”.* We feel that a 1 second simulation reasonably captures a burst of activity. Ensuring that Cl- reached a steady-state was not necessarily the aim of the simulations. For example, as the reviewer implies, for large GABA_A_ conductances, it is possible that it takes much longer than 1 s for Cl- to reach steady-state values. We felt that capturing the activity within a given, consistent timespan emphasises the effect of Cl- accumulation on firing rate within a short period of increased activity, instead of the maximum effect Cl- accumulation could have if given enough time to reach steady-state. Having said that, we certainly acknowledge that there may be further effects of dynamic chloride beyond 1s and so have provided an additional panel in Figure 5, which now demonstrates effects on firing rate after the simulations were run for 10 s. Additionally, we have plotted Chloride Index vs Inhibitory Conductance for different simulation durations in Supplementary Figure 1C and have included these different durations in the ΔEGABA vs Chloride Index population summary (Figure 6D).

*2) Cl concentration is taken as dynamical while other ionic concentrations are taken as constant. While in some cases this can be a good approximation, this assumption is ultimatey inconsistent with assumptions of constant osmolority and the almost electroneutrality of the intracellular space. Given the importance of intracellular potassium in particular, I suggest the author consider intracellular K+ as dynamically evolvoing in their model. A reasonable guess is that the sum of intracellular Cl- and K+ concentrations would be roughly constant. This would impact KCC2 tranport by making the impact of Cl increase on the rate of transport less important. Maybe this could be done just for a few simulations at least to estimate how large would the impact of fluctuating potassium concentration would be.*

We certainly agree with the author that ideally we would model dynamic changes in other ions as well, particularly K+. We have therefore implemented the reviewer’s suggestion and now include simulations where **“***the sum of intracellular Cl- and K+ concentrations would be roughly constant”* and use updated K+ concentrations to determine KCC2 transport rates*.* We now present this new data in Supplementary Figure 1D using the Chloride Index measure to note how the input-output function is altered under this condition.

*3) The authors adjust parameters so the mean firing rate is 5 Hz when each of the synapse receives input at 5 Hz. This seems reasonable, and seems to come from a steady-state assumption such as every neuron in a network firing at 5Hz. However this is not satisfactorily justified or referenced in the paper.*

We agree with the reviewer and have now added both a justification and further references in the paper. We now write “…until the neuron produced an output firing rate of 5 Hz to simulate a network with average firing rates in the theta frequency range (30,31)”.

References:

30: Buzsáki G, Moser EI. Memory, navigation and theta rhythm in the hippocampal-entorhinal system. Vol. 16, Nature Neuroscience. Nature Publishing Group; 2013. p. 130–8.

31: Losonczy A, Zemelman B V, Vaziri A, Magee JC. Network mechanisms of theta related neuronal activity in hippocampal CA1 pyramidal neurons. Nat Neurosci. 2010 Aug;13(8):967–72.

*4) In the discussion, the author refer to epilepsy. While the design of the paper is good is it stands, the impact of the paper could be increased if relevance of the results with respect to a particular pathology or physiological scenario would be more fleshed out.*

We thank the reviewer for this suggestion. We have now substantially added to the discussion where we discuss the relevance of our results to the initiation and spread of epileptic seizures (a particular pathology) and cortical up-states (a physiological scenario).

*Reviewer #2: In their computational study, the authors studied how chloride dynamics affects distal and proximal inhibition and how it modulates subtractive or divisive changes of the input-output function of neurons, which are driven by concurrent balanced excitatory synaptic drive. They found that spatial variations in chloride concentration can emerge despite uniform expression of chloride pumps. In addition, they observed that whereas chloride accumulation in dendrites diminishes the ability of distal inhibition to trigger subtractive shifts in the input-output curve, the ability of proximal inhibition to trigger divisive changes of the input-output curve remained largely unaffected by dynamical chloride. The methods are well described and the results are appropriately interpreted.*

*Major points:*

** The authors show convincingly that concurrent inhibitory and excitatory synaptic drive may lead to spatial variations in chloride despite uniform expression of chloride pumps. However, experimental data show that the expression of chloride pumps may not be uniform leading to a dendro-somato-axonal gradient in EGABA with chloride concentration becoming progressively higher toward soma (Gulyas et al. Eur J Neurosci 2001, Szabadics et al. Science 2005, Khirug et al. J Neurosci 2008). The authors should explore such scenario in their model – e.g. by making the KCC2 pump strength spatially inhomogeneous with a higher expression in dendrites/soma and low/zero expression in the axon. Can enhanced KCC2 pump strength in dendrites counteract the inhibition-compromising effects of dendritic chloride accumulation?*

We thank the reviewer for providing excellent references with regard to experimental demonstration of subcellular differences in EGABA and the inhomogeneous expression of chloride cotransporters. We have now included these references the manuscript, except for Szabadics et al. because we have not included data where we lowered the KCC2 expression in the axon (although we ran the simulations, they provided little further insight beyond the already plotted distal KCC2 strength). In addition, following the reviewer’s suggestion we have now run simulations using our model with regimes where the “expression” or activity of KCC2 is inhomogeneous, ie different in the distal dendrite as compared to the proximal dendrite and soma. Again we have used the Chloride Index metric to explore how Cl- dynamics effects input-output curves. In further simulations, we demonstrate the reduced but still substantial effect of Cl^-^ accumulation in dendrites even with 400% KCC2 expression in the distal dendrites (the region of input) as compared to other regions (see Supplementary Figure 1). For a more general effect, Figure 6B (Chloride Index vs Inhibitory Conductance for different KCC2 Strengths) indicates that even homogenous expression of increased KCC2 strength does not fully counteract the effects Cl- accumulation for cases of large inhibitory synaptic drive.

** Line 155: “Combined with mechanisms to describe Cl- influx via both tonic and synaptic activation of GABAARs…” In Methods, the authors describe the synaptic models of phasic GABA-A responses. Did they include also tonic GABA-A receptors/conductances in their models? I could not find the description of them. They probably mean tonic chloride currents via leak chloride channels. This has to be clearly distinguished and described in the paper.*

We apologise that this was unclear. The reviewer is quite correct, we do mean tonic chloride currents via leak chloride channels. We have amended this sentence accordingly: “Combined with mechanisms to describe Cl- flux via both constitutively active Cl^-^ leak channels and synaptic activation of Cl^-^ permeable GABA_A_Rs”

** Line 582-583: The authors report that they modelled excitatory synapses as voltage-dependent NMDA conductances. Were AMPA synapses not included? If not, why? Mixed AMPA/NMDA synaptic models would be biologically more realistic than pure NMDA inputs. Recent compartmental modelling of chloride concentration changes used stochastic AMPA/GABA-A inputs (Lombardi et al. Front Cell Neurosci 2018, Lombardi et al. IMJS 2019). Are the results of the current paper in line with this modelling or are there some important differences when NMDA synapses are included?*

We thank the reviewer for pointing out this limitation of the initial model. All simulations with NMDA synapses have now been performed again, but now with mixed AMPA/NMDA synapses. We have used the same model of excitatory synapses as the Blue Brain Project (Ramaswamy et al. J Physiol 2012). Our findings relating to the effect of dynamic Cl- on Cl- homeostasis and the input-output function of neurons remain the same. However we do note that for balanced synaptic input at higher frequencies, firing rates are different, particularly for proximal inhibition (see new Figure 3).

** Lombardi et al. IMJS 2019 showed that membrane resistance (determined by gpas) critically influences the amount of chloride changes. Is that the case in the authors’ model? Do their conclusion hold for a wide range of gpas values? (Modeling in the paper was performed only for a selected input resistance of 350 Mohm.)*

Following the reviewer’s suggestion we have now repeated simulations with different membrane resistances by adjusting ionic leak conductances whilst maintaining the same ratio of K+:Na+:Cl- conductances. We could then explore the effects of dynamic Cl- on the input-output function using the Chloride Index metric at different membrane resistances, which generate different input resistances as “recorded” from the soma. We note that different input / membrane resistances certainly impact output firing rates for different synaptic input, however the effect of Cl- dynamics on the input-output function remains the same and is dependent on changes in EGABA (see new Figure 6 and Supplementary Figure 1).

** Prescott, Sejnowski and De Koninck (Mol Pain 2006) showed that inhibitory regulation of firing can be compromised by decreasing hyperpolarization and/or shunting. The authors should discuss their results in the light of this paper/ these findings.*

We thank the reviewer for pointing us to this important early modelling study on how the changes in Cl- reversal potential impact the efficacy of inhibition and firing rates. We now briefly discuss and reference this paper in the discussion section.

** Figure 4 illustrates subtractive and divisive effects of distal and proximal inhibition, respectively. Subtractive inhibition should result in a rightward shift of the IO curve without affecting its slope whereas divisive inhibition should alter the slope of the IO curve. However, in the figure it seems that distal inhibition affects the slope, whereas proximal inhibition shifts the curve and only minimally affects the slope. Can authors comment on this?*

We agree with the reviewer that previously the subtractive and divisive effects were not obvious. We have now re-run the simulations with AMPA/NMDA mixed synapses and importantly have plotted a wider range of synapse numbers to more clearly demonstrate the effects. Previously we also fitted the results to a sigmoid curve, but have now plotted the raw data points. The new Figure 4 now very clearly shows the subtractive effects of distal inhibition and the divisive effects of proximal inhibition in our model.

*Minor points:*

** Figure 5: Replace “Excitatatory” by “Excitatory” (twice).*

We apologise for this error which has been corrected.

** Line 265: Add “blue”*

This has been added.

*Reviewer #3: Major concerns:*

*The study examines the effect of dynamic changes in [Cl]i on neuronal input-output relationship. Although there are some interesting aspects in this work, much of the results are intuitive and lack significant insight to be gained from a modeling study. For example, it is expected that the effect of inhibition on input-output function is weaker when inhibition is inter-mixed with excitation. Similarly, if [Cl]i is dynamically changing, any time delay in these changes could minimize the effects on the input-output relationship when excitation/inhibition are colocalized (Fig 4B). Most importantly, the model does not seem to reproduce the divisive effects of proximal inhibition on input-output relationship (Fig. 4D). This limits the confidence in the model itself. Model innovation is minimal. Overall, the results are not significant to improve our understanding of inhibitory effects on input-output processing in neurons.*

We acknowledge Reviewer 3’s criticisms.

Concerns relating to novelty: We agree that “*if [Cl]i is dynamically changing, any time delay in these changes could minimize the effects on the input-output relationship when excitation/inhibition are colocalized*”, indeed, this is what we show in Fig. 5. However we feel that the change in the input-output properties of neurons during ongoing synaptic drive due to Cl- dynamics is not well appreciated in the field and is therefore an important contribution. Secondly, we develop a novel metric to quantify the effects of dynamic Cl- (the Chloride index). We use this metric to show that although various parameters (Cl- cotransport, dendritic diameter, input resistance etc) effect output firing rates, actual changes in the input-output properties of neurons due to Cl- dynamics are entirely mediated by changes in dendritic EGABA. Our paper offers various other insights including explanations for spatial variations in Cl- concentration as well as differences between proximal and distal inhibition in terms of sensitivity to the effects of Cl- dynamics.

Concern relating to the model reproducing the divisive effects of proximal inhibition: We are very grateful that the Reviewer raised this concern as it has encouraged us to conduct a thorough re-analysis of the model’s ability to capture classic subtractive and divisive gain effects. This has led us to improve the model by incorporating Hodgkin-Huxley kinetics in the axon and to perform comprehensive repeats of our simulations, with new synaptic models, and over a wider range of synapse numbers (see for example new Figure 4). In addition, we plot the raw data points. We thank the Reviewer for encouraging us to do this and we believe it is now very clear that proximal inhibition has a divisive effect in our model.

Collectively we believe this work does improve our understanding “of inhibitory effects on input-output processing in neurons”.

*Minor comments:*

*Lines 85: “inputs can perform a divisive operation by changing the slope (gain) of the input-output function, or” What is the direction of this “change” – be specific.*

We have replaced the word “changing” to “reducing” to be more specific.

*Lines 97 – 99: “Intracellular Cl- concentration ([Cl-]i), and hence EGABA, can differ between
subcellular neuronal compartments, which has been suggested to result from spatial differences in the function or expression of cation-chloride cotransporters in the neuronal membrane (10–12).”*

*Clarify the above statement.*

In order to clarify this statement we have added another sentence following it. “That is, synaptic activation of GABAARs can have different effects on the neuronal membrane potential because of differential expression of Cl- homeostatic mechanisms”.

*Lines 110 – 113: “Whilst the vast majority of theoretical models of inhibitory signalling and neuronal computation assume static values for EGABA, previous studies have addressed the biophysical underpinnings of Cl homeostasis and ionic plasticity in neurons (20,22), the effects of neuronal morphologies on Cl accumulation (19,21,23), and how dynamic Cl- affects neural coding (24).”*

*Please summarize briefly what these studies suggest on Cl- dynamics rather than simply stating that Cl- dynamics has been explored.*

As suggested by the reviewer we have now added very brief summaries in this introductory paragraph to provide more context for the studies referenced.

*Lines 135 – 141: Please clarify the rational for using organotypic slices as a suitable preparation to measure Cl-extrusion. These sentences are too long and should be restructured for readability.*

We have now added the following sentence where we clarify the use of organotypic slices for measuring Cl- extrusion:

“Organotypic hippocampal brain slice possess mature Cl- homeostasis mechanisms, as evidenced by their hyperpolarizing EGABA (40,41) and the fact that KCC2 is the major active Cl- transporter in these neurons (42).”

In addition we have restructured the following sentences which we agree were too long.

*Lines 177 – 180: The sentence begins with “Extensive evidence….”, but there are no relevant citations provided. Clarifying what type of methods and preparations revealed differential [Cl]i in those experimental studies would also be pertinent.*

We now add references to 3 studies, which show differences in dendritic versus somatic Cl- concentrations as well as the methods used to measure Cl- in each case.

*Line 224: It may be better to call “synaptic structure” as “synaptic configuration” here and elsewhere.*

We agree with the reviewer and have changed “synaptic structure” to “synaptic configuration”.

*Lines 235 – 237: This sentence is too long and cryptic. Please explain clearly.*

We agree with the reviewer and have broken the sentence and have attempted to explain what we mean more clearly.

*Lines 243 – 244: “predicted” must be replaced with “speculated”*

We have made the replacement as suggested.

*Lines 202 – 222: Some of the descriptions provided here in the Figure legend (e.g., balanced input) can go in the main manuscript. Figure legends must be very brief only describing the graphic components of the figure.*

In this figure legend we have attempted to keep the legend as brief as possible whilst describing the graphic components as well as what they show.

*It would be useful to provide a figure panel showing the time series of [Cl]i in the model in response to balanced input with different synaptic configurations.*

On the reviewer’s suggestion we now provide figure panels for the new Figure 3 where we show “the time series of [Cl]i in the model in response to balanced input with different synaptic configurations”.
