# Supplementary material for: Chloride dynamics alter the input-output properties of neurons
Source: PLoS Comput Biol. 2020 May 26;16(5):e1007932. doi: 10.1371/journal.pcbi.1007932 (PMC7307785; doi:10.1371/journal.pcbi.1007932)
Supplement: Attachment — Submitted filename: Response_to_reviewers (Currin et al).docx [file pcbi.1007932.s004.docx]

Response to reviewers

We thank the editorial team and the reviewers for their positive reviews and their suggested corrections, which we have addressed below.

*Reviewer #1: I feel the authors have satisfactorily answered the comments of the reviewers and that the current version of the manuscript is worthy of publication as it is.*

*A few minor comments:*

*1) At several places, when the authors write Cl- and K+, the - and + are not in exponent format. Please verify.*

We apologise for these typos and have now ensured that exponents are used throughout the document.

*2) I'm still uneasy with p values such as 10^(-140). Is it different from 0 then? Is it within some numerical uncertainty? Or if the p-value is so small, can we say that something is certain? Also, just above the discussion sectio, -140 should be in exponent*

On the reviewers suggestion we have now changed the p value statement to read: p < 0.00001.

*3**) Page 19, line 560, what are ' mature and stable Cl- homeostasis mechanisms'?*

We have now simplified this to read “mature Cl- homeostasis mechanisms”. What we mean by this is that neurons in organotypic brain slice cultures are developmentally mature neurons with a hyperpolarized EGABA and significant KCC2 activity (as opposed to developmentally immature with more depolarized EGABA and relatively high NKCC1 activity).

*4) line 579,* *is it mOsm or mOsM?*

We apologise for this error. It has been corrected to mOsm.

*5) Line 606, could the authors specify what is the meaning of (\xi=1)?*

We have removed direct reference to ξ and instead have added a sentence about ‘f-in’ synapses that encapsulates the original meaning of this term (that the interval of spikes is negative-exponentially distributed when the process has a noise term [ξ] of 1).

Reference 58 is The Neuron Book

*6) Figure 1 c, the units are written as (1/mMs) which can be misleading. Would it possible to write mM^-1 s^-1 or (1/(mM\cdot s))?*

We agree and have corrected to (1/(mM⋅ s))

*Reviewer #2: The authors have addressed all my concerns. Minor points:*

** A new important experimental study on dendritic inhibition has recently been published - the authors might want to cite it: https://www.nature.com/articles/s41467-019-13533-3*

We thank the reviewer for alerting us to this important paper which we now reference in our discussion.

** The authors performed new simulations with manipulations of ionic leak conductances and the associated input resistance (Fig. 6 & S1A). However, they don't mention the rationale or the motivation for it - namely previous results of Lombardi et al. https://www.ncbi.nlm.nih.gov/pubmed/30897846 For a better understanding of the background for these simulations, this could be mentioned at the beginning of the relevant results section.*

Thank you for this suggestion, we have now included a sentence to provide rationale (and a reference to Lombardi et al.) for the further manipulations we have performed

** L. 464 - replace "stength" by "strength"*

This has been corrected.

*Reviewer #3: The authors have made significant changes to the manuscript since last review which has greatly improved the manuscript and satisfactorily clarified much of the reviewer concerns. However, there are a few minor points to be addressed as noted below:*

*1) Ref Fig 2 and other related simulations. Please provide the stdev of the input firing rates and note whether it differs for different mean input firing rates (e.g.., mean rate of 5 Hz versus 20 Hz).*

We have now indicated in the methods section that input firing rates have a standard deviation of the √ of the mean.

“Each f-in synapse received a $\mathrm{mean}\pm\sqrt{\mathrm{mean}}$ (standard deviation) frequency with spike time intervals sampled from a negative exponential distribution (58).”

Reference 58 is: The Neuron Book

*2) Line 414: As per the illustration in Fig 6A inset, the authors have to spell out x50 as the relative excitatory conductance at half-maximal firing rate in the results section. Currently it seems like they call the half-maximal firing rate as x50 which is not the case.*

We apologise that this is not clear we have now corrected the “x50 definitions” as follows

“the relative excitatory conductance at the half-max firing rate, $x_{50}$”

We have also made a slight adjustment to Fig 6 to indicate how the y-values were chosen

*3) Figure 3, panels A and B: Please change Y-axis label to Firing Rate (Hz).*

We have now changed the label as suggested.

*4) Line 513: Previous work such as Venugopal S, J Neurophysiol, 2011 has to be cited.*

Their work has now been cited as part of the following edited sentence

“This also confirms previous modelling studies that show how shifts in EGABA compromise the inhibitory control of firing rates and, in turn, suggest the targeting of Cl^-^ extrusion mechanisms and GABA_A_ receptors as part of therapies for neuropathic pain and spinal cord injury (47,48)”

*Line 189 Remove "well" or provide multiple references*

We have removed “well”.

*Line 206 Begin sentence with "Note that multiple candidate..."*

This sentence has now been amended as suggested.

*Line 207 Remove extra period*

The extra period has been removed.
